# Surveillance of drug prescribing: why outliers miss their targets – a qualitative study

**DOI:** 10.1186/s12913-024-12189-0

**Published:** 2025-01-03

**Authors:** Julia Gollnick, Nikoletta Zeschick, Franziska Hörbrand, Peter Killian, Maria Sebastiao, Thomas Kühlein, Norbert Donner-Banzhoff

**Affiliations:** 1 Institute of General Practice/Family Medicine, Philipps-University of Marburg, Karl-Von-Frisch-Straße 4, 35043 Marburg, Germany; 2 Institute of General Practice, Friedrich-Alexander-University of Erlangen-Nürnberg (FAU), Universitätsstr. 29, 91054 Erlangen, Germany; 3 Association of Statutory Health Insurance Physicians, Elsenheimerstraße 39, 80687 München (Bavaria), Germany

**Keywords:** Drug prescriptions, Quality of health care/economics, Qualitative research, Drug costs, Clinical decision-making

## Abstract

**Background:**

Rising costs are a challenge for healthcare systems. To keep expenditure for drugs under control, in many healthcare systems, drug prescribing is continuously monitored. The Bavarian Drug Agreement (German: Wirkstoffvereinbarung or WSV) for the ambulatory sector in Bavaria (the federal state of Germany) was developed for this purpose. Physicians must reach defined drug target quotas for prescribing generic drugs and certain recommended drugs specified and measured with defined daily doses (DDD). A subgroup of physicians, known as outliers, may miss their drug targets. The objective of this qualitative study was to understand the reasons physicians miss their targets.

**Methods:**

We identified outliers based on drug prescribing data from the association of statutory health insurance (SHI)-accredited physicians (KV). Outliers were invited to participate in semi-structured interviews.

**Results:**

Out of 401 outliers thus identified *n* = 26 physicians were interviewed. Their prescribing behaviours are affected by competing demands regarding drug decisions, such as saving staff time, costs, and discussions with patients. Often, their freedom to prescribe is limited by previous prescribers. Ease of administration of drugs not recommended also plays a role. Uncritical enthusiasm regarding the effectiveness and safety of drugs with recommendations, often reinforced by pharmaceutical marketing, leads to missed targets. Some physicians have coping strategies to avoid becoming outliers.

**Conclusions:**

Investigating physicians not meeting their targets helps us understand beliefs and barriers for appropriate drug prescribing. Based on these kinds of findings, surveillance procedures can be improved, and physicians can receive support to meet targets in the future.

**Trial registration:**

This trial has been registered in the German Register of Clinical Trials (DRKS: DRKS00016161; registration date 07. December 2018).

**Supplementary Information:**

The online version contains supplementary material available at 10.1186/s12913-024-12189-0.

## Keypoints

Regulating drug prescribing aims to control costs for healthcare systems. In fact, some physicians miss their drug targets. Reasons are individual, but mechanisms can be identified.

**Supplementary Information:**

The online version contains supplementary material available at 10.1186/s12913-024-12189-0.

## Background

Rising costs for prescribed drugs are a challenge for all healthcare systems [1]. To limit rising costs for drugs, the German law (Social Security Code, §12 SGB V) states that drug therapies must be sufficient, appropriate, and economical and must not exceed what is necessary. Patients cannot not claim medical services that are uneconomical or unnecessary, and physicians may not provide those services [2]. In Germany, all drugs with market authorisation when entering the market are covered by the statutory health insurance funds (SHI). The only restriction applies to the price, which depends on the additional benefits of a drug in comparison with established competitors and is stated by the Joint Committee (Gemeinsamer Bundesausschuss – G-BA). The idea is to make innovative drugs conferring relevant benefit available, while discouraging brands with high cost but negligeable or lacking advantage over established treatments [3]. It still results in large numbers of drugs (brands) being covered by the SHI, leaving decisions and related economic considerations to prescribers. The Associations of the SHI-accredited physicians (KV) is responsible for the communication with prescribers and arbitrates the interests of SHI and physicians regarding drug prescribing and prescribing costs.

Surveillance procedures have been developed for the ambulatory sector to monitor prescribing costs. They are negotiated between the SHI and KV, and comprise targets, quality criteria, cut-offs etc. [4]. Physicians who do not comply with their prescribing targets obtain individualised feedback but may ultimately suffer financial sanctions [4].

In Bavaria, a state within the Federal Republic of Germany, a novel agreement (Bavarian Drug Agreement, German: Wirkstoffvereinbarung, in following WSV) was introduced in 2014 [5, 6]. While other states focus on absolute costs for prescribed drugs, the WSV requires the prescription of preferred drugs [7]. The subject matter of prescriptions are DDDs [7], which present the average maintenance daily dose for adults (WHO) [8]. The number of DDDs contained in each pack is calculated according to the number of tablets and the amount of drug. In the WSV 2.0, for 31 indication areas, prescribing targets consisted of proportions prescribed for recommended drugs or generics [9]. For example, orthopaedists must prescribe 63% (WSV 2.0) defined daily doses (DDDs) of generics in ‘drugs for the treatment of bone diseases’ [10]. ‘Recommended drugs’ are usually well-established drugs supported by evidence regarding their safety and effectiveness. They are recommended by current guidelines and frequently available as generics or biosimilars. In 2018 and 2019, for example, when conducting our interviews with gastroenterologists, 39% of their prescribed DDDs within ‘TNF-alpha-inhibitors’ had to be for the following four recommended drugs: Benepali® = etanercept, Flixabi® = infliximab, Inflectra® = infliximab, and Remsima® = infliximab [9, 10].

The proton pump inhibitor (PPI)-drug target restricts the amount of prescribed DDD per patient. Therefore, physicians have limited PPI-DDD (20 mg omeprazole = 1 DDD) per patient. The number per patient is an average number, so higher DDD numbers for one patient can be levelled by narrow DDD numbers of other patients.

Targets are adapted to specialisation groups [10].

**Fig. 1:**
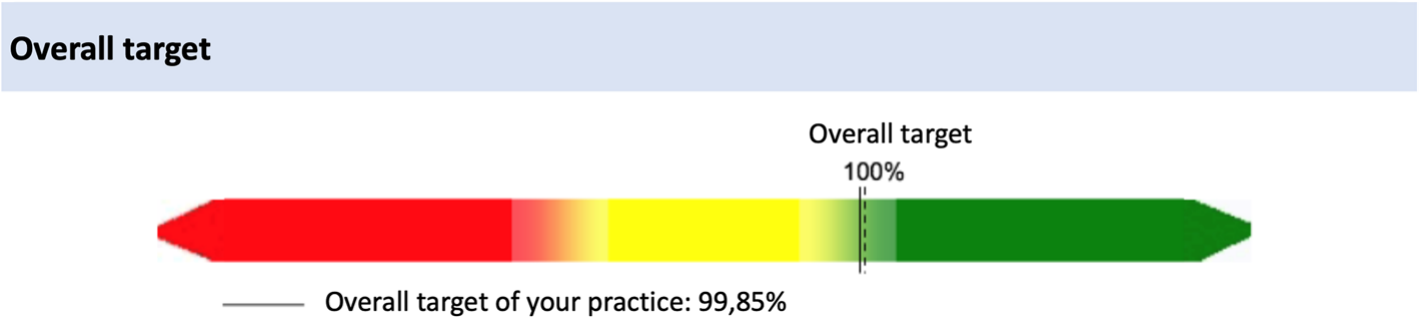
Feedback regarding overall targets—modified according to the KV

Every quarter, physicians receive feedback by the KV on whether and to what extent prescribing targets have been met. Additionally, all targets are weighted according to the prescribed DDD-volume and various factors (e.g., costs and additional benefits) and are cumulatively calculated as a so-called overall target covering all drug prescriptions issued by a particular practice (Fig. 1).

Despite the detailed feedback mechanism and the potential threat of financial sanctions, some physicians fail to reach their overall target in the long term.

As part of the project *WirtMed—the prescription of medicines: Audit and Control of Efficiency and Quality* funded by the Innovation Fund of the Federal Joint Committee (G-BA), we focussed on physicians repeatedly missing their prescribing targets. The aim was to understand the prescribing behaviour of these physicians by exploring associated beliefs, reasons for prescribing drugs, which are not recommended, and barriers against compliance with the WSV and coping strategies in a qualitative study.

We already reported on indicators to be used for prescribing surveillance aiming cost control based on prescription data [11].

## Methods

For this study, we used a qualitative design to explore this research area and generate hypotheses for missing drug targets. We conducted semi-structured interviews with physicians who missed their prescribing targets during the observation period from the second quarter of 2017 to the first quarter of 2018. These outliers were defined as:

1. less than 85% overall target achievement under the WSV, and
2. less than 3% improvement regarding the overall target achievement in two sequential quarters.

Physicians meeting these criteria were identified by the Bavarian KV (KVB). In Germany, there are physicians of different specialisation groups in ambulatory care, usually without hospital affiliations. Therefore, we did not restrict invitations to any primary or secondary care specialisation groups. Physicians thus identified received a letter from the KVB, which included an invitation from the Philipps-University of Marburg (Department of General Practice) and Friedrich-Alexander-University of Erlangen (Department of General Practice) to participate in the study. If interested, physicians were asked to contact the Department of General Practice of the Philipps-University Marburg to arrange an interview. The KVB thus received no information on who participated in the survey.

JG (female, pharmacist) developed an interview guide covering participants’ practice philosophy, relationship with their patients, prescribing behaviour, professional learning and staying up-to-date, experiences with the WSV, and opinions on prescribing surveillance (see supplement, S1: interview guide). JG and NL (female, social scientist) pretested the interview guide with physicians of the research teams in Marburg and Erlangen. The interviews started with more general topics and gradually approached the contentious area of drug prescribing. JG and NL jointly conducted the interviews. The interviews took place in participants’ practices between December 2018 and July 2019 and were conducted in German. JG made fieldnotes (including experiences and impressions) during and after the interviews, which were subsequently discussed between JG and NL. The interviews were recorded and transcribed by an external service provider. JG coded the transcripts using MAXQDA. JG developed a code tree, which was continuously modified in discussion with another researcher who in parallel coded 13 interviews. With the improved tree JG coded the second 13 interviews, resulting in only very little further modifications of the code tree. The resulting final code tree was used by JG for the final coding of all 26 interviews. We used an inductive-deductive procedure, which is oriented towards the qualitative content analysis, according to Mayring and Fenzl [12]. Deductive codes evolved from the interview guide, and inductive codes resulted from the contents of the interviews. Coding and data analyses started after conducting the first interviews. We presented and discussed results continuously to the research team. For choosing the quotations for the result section we first built the main massage of the single code and then identified the most pertinent citations. We undertook further selection according to brevity and fit with other utterances. The authors translated the German quotations by participants into English. A professional service undertook language editing of the paper with additional checks of quotations by authors.

We obtained permission from the ethics committee of the Philipps University of Marburg (Study 118/18, 05.09.2018). This part of the subproject of the WirtMed study was registered with the German Register for Clinical Studies (DRKS-ID DRKS00016161; registration date 07. December 2018) [13].

In the following, we mentioned brand names of drugs only if they were an explicit topic of participants’ adherence. We present original quotations with the following coding: TPD1819-XY (TPD = subproject D (German: Teilprojekt D), 1819 = interviews conducted in 2018 and 2019, XY = number of interview).

## Results

### Study sample

The KVB identified a total of 401 practices out of about 13,600 in Bavaria as failing to meet their targets and sent them invitation letters. 34 responded and gave their consent to take part in the study. Six dropped out because of lack of time or interest. 28 interviews were scheduled, and we conducted 26 of those (length of time 30 to 105 min). The research-team decided to cancel the last two interviews (number 27 and 28; specialisation group: orthopaedist) due to reaching the theoretical saturation from repeating the contents of former interviews (challenging drugs and reasons for prescribing were the same or very similar in all interviewed orthopaedists; we therefore did not expect any additional findings from the two interviews with this already well-represented specialisation group). The sample was composed of physicians of the characteristics and specialisation groups listed in Table 1.

**Table 1:** Description of the study sample

Characteristics		Number of Participants
**Age**	41–50 years	6
	51–60 years	17
	> 60 years	3
**Sex**	Male	21
	Female	5
**Practice Location**	City	5
	Smaller city	18
	Countryside	3
**Specialisation Group**	Cardiologist	1
	Child and youth psychiatrist	1
	Gastroenterologist	2
	General practitioner	3
	Haematologist and Oncologist	2
	Neurologist	2
	Neurosurgeon	1
	Ophthalmologist	1
	Orthopaedist	9
	Paediatrist, sub-specialisation rheumatology	2
	Surgeon	2

Total number of participants: *n* = 26

Smaller city: < 100.000 inhabitants

City: > 100.000 inhabitants

### Qualitative findings: prescribing practice

Most of the interviewed physicians were unhappy about being an outlier, which the interviewers recognised due to emotional and/or intense speech of the participants (special emphasis shown in capital letters). There are different mechanisms influencing the prescribing behaviour resulting in being an outlier (Table 2).

**Table 2:** Mechanisms influencing the prescribing behaviour of outliers (no defined order)

Mechanisms effecting the prescribing behaviour of outliers
Competing demands
Limited decision latitude
Adequacy of indicators
Lack of knowledge regarding WSV
Faulty beliefs
Ease and safety of application
Coping strategies
Medical information management

These mechanisms we present more detailed in the following.

#### Competing demands

Patients asking for drugs and/or manufacturers (brand instead of generic) often leads to a breach of cost-effectiveness principles: *‘One prescription […]doesn’t cost me any nerves in the total bill. But it costs me a quarter of an hour of hoarseness to explain to the patient why […] [I] will NOT prescribe him this drug’* (TPD1819-14). Physicians explain being tired of this kind of discussion with their patients and try to avoid them by acquiescing to patients’ demands.

Participants report having to change the drugs they have prescribed over years also represents a particular challenge. They state, having to explain that another drug is preferable puts themself in a contradictory position. In these cases, physicians feel that their competence is contested. This feeling seems to be particularly strong with biosimilars, which according to the WSV, have to be prescribed preferentially. Participants criticise, first they told their patients why one drug (brand) was right for them, and now they must explain why another drug (biosimilar) is appropriate.

Participants mention patients regard cheaper drugs as being of poorer quality. They also try to convince their patients that this is not the case, but they are not always successful. A few patients even change to another practice to obtain prescriptions of their preference.

Some physicians did not meet the oral anticoagulant target because they prescribe direct oral anticoagulants (DOACs) too frequently instead of vitamin-K-antagonists (VKA). They stress the ease of administration and fewer visits for blood monitoring, thus saving staff time and costs. Participants also mention to save staff time and costs by prescribing the biological Humira® (adalimumab) for inflammatory bowel disease (IBD) because it can be administered by the patient at home (see ease and safety of application).

Thus, physicians feel their competence is challenged if they change drug therapies to meet prescribing targets. Their acceptance by their patients and the economic viability of their practice are at stake, which has a relevant influence on prescribing behaviour.

#### Limited decision latitude

Prescribing drugs after initiation by other physicians is a reason for not following the WSV. Participants state, patients often ask them for repeat prescriptions that do not conform to prescription targets: *‘It must have been a patient who got it from someone else for some reason, and I said, “Okay, then you’ll get it from me, too”’* (TPD1819-08). In this case, physicians explain not to question the decisions made by their colleagues.

In addition, physicians mention being uncertain whether switching to biosimilars might cause the risk of litigation in case of side effects or complications. Some state, for example, that although the structure of the protein is the same, the preparation is not always identical regarding its composition and further ingredients.

Some participants report that patients with special management problems are referred to them. They explain advanced or more severe illness often requires the prescription of second or third-line drugs, which are usually not in line with the requirements of the WSV.

#### Adequacy of indicators

The participants criticise that the addition of new drugs to the list of recommended drugs often happens only after long delays. They state that the preference of well-established and cost-effective drugs prevents patients from obtaining new and, in participants’ view, more effective or safer drugs. For a long time, the WSV stipulated the prescription of VKAs instead of DOACs, and only a few DOACs (e.g., apixaban and edoxaban) were added to the list of recommended drugs. However, a certain percentage of VKA prescriptions is still required. Participants maintained that the WSV is lagging with regard to the accommodation of new drugs.

Some physicians recognise there are conflicts between guidelines and preferred drugs within the WSV. They mention drugs for multiple sclerosis (MS) and obsessive–compulsive disorder in children as examples. Participants cover, in this case physicians who e.g., have a sub-specialisation for MS run the risk of prescribing recommended drugs not frequently enough.

#### Lack of knowledge regarding WSV

Participants are familiar with the WSV, but detailed knowledge about the different drug targets is sometimes missing. For example, some physicians seem to be confused about handling drugs covered by discount contracts by the insurance of a particular patient. They describe, at first, they had chosen drugs covered by discount contracts, but after the expiration of a discount contract, they risk missing a target if the drug is neither generic nor preferred. So, physicians state, if they prescribe drugs with discount contracts, which they are required to do, this could, in some cases, lead to missing the drug targets of the WSV. As a result of these changes, participants lost track of the various details and applicable regulations for single drugs, and discount contracts are part of the confusion.

Furthermore, participating physicians state, they had other PPI-equivalent dosages in mind than the WSV requests, such as 40mg pantoprazol, assumed to be equivalent to 20mg omeprazol. They explain, in contrast the WSV defines 1 PPI-DDD being equivalent to 20mg omeprazol or 20mg pantoprazole. In fact, this is important because the target refuses 2 DDD for PPI prescribing.

Most of the interviewed physicians mention helplines and advice by the KVB to clarify issues regarding the WSV, resulting in improvements in reaching their targets.

Some participants experience the WSV as a very complex surveillance procedure, and some do not understand all the requirements. For example, one reports he is not familiar with the concept of DDD. Some physicians have not grasped the fundamental difference between the WSV and the previous surveillance policy. Financial budgets were calculated for the latter, which practices were allowed to exceed only if their practice had special characteristics. Those participants seem not to know that for the WSV, budgets are no longer calculated and are replaced by targets measured and specified by DDDs.

#### Faulty beliefs

Patients with osteoporosis should preferably receive generic drugs, and bisphosphonates are available in generic form. However, most of the participating orthopaedists state missing their prescribing target by often preferring denosumab (only available as Prolia®). These physicians consider Denosumab to be *‘actually almost unrivalled’* and *‘a real advance’*. They feel that it prevents bone loss and promotes bone formation, while bisphosphonates only prevent further bone loss. Furthermore, they mention very low bone mineral density (BMD) as an indication for choosing denosumab for its better ‘mechanistic evidence’. Likewise, because of the limited duration of therapy for bisphosphonates, physicians feel the necessity of switching to another drug.

#### Ease and safety of application

Guidelines recommend Adalimumab (at the time only available as Humira®) and infliximab for treating IBD. The WSV, however, recommends only infliximab biosimilars. Participants justify preferring adalimumab for IBD instead of the biosimilar infliximab with patient comfort due to independent subcutaneous application at home, while infliximab is administered intravenously: *‘[Humira® is] Much more comfortable, because they don’t have to come to the practice every six or eight weeks and be monitored for four hours, they just do it themselves. The effectiveness is about the same, maybe even a little better here and there.’* (TPD1819-02).

One participant experienced several patients with severe anaphylactic reactions after infliximab infusions (no further specification of whether biological or biosimilar, the argument was just related to the antibody infliximab), which is why he prefers prescribing adalimumab due to the better risk profile: *‘[adalimumab is] from the way of production NOT so allergenic or actually not allergenic at all. Therefore, much less risky for the patient.’* (TPD1819-02).

While missing the drug target ‘drugs for the treatment of bone diseases’, some physicians perceive the subcutaneous application of denosumab every six months as an advantage compared to weekly oral bisphosphonates. Further participants reason the therapy with denosumab with the possibility of avoiding gastrointestinal side effects such as oesophagitis.

#### Coping strategies

To avoid becoming an outlier, participants have developed coping strategies. For example, some physicians prescribe drugs conforming to targets themselves but refer patients in need of other drugs elsewhere. Accordingly, physicians accepting these referrals complain: *‘[…] then I’m more likely to be the one who is referred to, because if it’s approved and there’s no option, then I write the prescription.’* (TPD1819-03)*.* As a result, because of a high proportion of uneconomical prescriptions, they describe becoming an outlier. One physician reports that he would treat patients and apply the injection but before he sent his patients to other physicians to obtain a prescription. Sending to out-patient care units of hospitals is another mentioned way of avoiding drug prescriptions which are not recommended by the WSV. Another participant states some drugs, for example, PPI in addition to NSAID therapy preventing gastrointestinal-side effects, are prescribed privately to be paid for by patients themselves to avoid missing the prescribing targets.

#### Medical information management

Almost all participating physicians report being regularly visited by pharmaceutical representatives. They regard pharmaceutical representatives as an important source of information for new drugs and medical devices.

Most participants are happy to receive advice from the Bavarian KV (print material or in person). However, they often discovered that letters or messages had gotten lost, which may have contributed to their incomplete knowledge of the WSV.

## Discussion

Participants are missing their prescribing targets for a variety of reasons. When prescribing, they have to accommodate the evidence base of available drugs, health economic prioritisation, influence by pharmaceutical manufacturers, their patients’ expectations, and the norms of their profession. Specific behaviours, such as prescribing drugs not recommended or missing generic targets, pose a risk to become an outlier. These are associated with beliefs, norms and conflicting expectations. Some of these reflect true dilemmas, others, however, result from misunderstandings, lack of information or values not consistent with the WSV. In the following, we discuss the reasons mentioned and their justification and set them in a scientific, economic, and political context.

### How do prescribers become outliers?

#### Competing demands

Patients’ demands influence physicians’ behaviour, especially requests for specific drugs and tests, which was also shown in different studies, for example, in a qualitative study among patients in primary care [14] or oncology [15]. Not all of these are to be preferred from a medical or cost-effectiveness perspective. Tactics to deal with problematic patient requests should be disseminated to make prescribers more effective in this regard.

Participating physicians prefer DOACs to VKAs to avoid complex monitoring and, thus, to reduce their costs, which is also shown as a benefit of DOACs in other studies [16]. This is also the case when prescribing adalimumab, which, in contrast to infliximab, does not require a longer stay in the practice and follow-up after the infusion, as is the case with infliximab [17, 18]. In Germany, practices are privately owned. By prescribing drugs and saving staff time, which is not preferred from the WSV perspective, physicians can thus shift costs to the insurance covering more expensive drugs [1].

#### Limited decision latitude

Frequently, physicians are confronted with requests for the continuation of a prescription that they have not initiated themselves. The more treatments for chronic disease are available, which must be prescribed continuously, the more frequently this situation arises. Also, the fact that German primary care physicians cannot control their patients’ access to secondary care (absence of gatekeeping), puts a central assumption of the WSV in question, i.e., that each prescriber has full control over their prescribing. Since hospital physicians are not subject to the WSV, they often prescribe drugs, leading to conflicts with the WSV once the patient has been discharged to ambulatory care. This intersectoral challenge was also found in another subproject of the WirtMed project [19]. This is a deficiency of all systems surveying drug prescribing in Germany.

Other authors demonstrated physicians’ prescribing behaviour to be influenced by fear of litigation [20, 21], especially switches to biosimilars [22]. That’s one reason for not switching biologicals to biosimilars and preferring biologicals. While our interviews were conducted, the context for this decision changed. In 2018, a phase III study showed the equivalence of adalimumab biological and biosimilars regarding efficacy, safety, and immunogenicity in patients with psoriasis, where adalimumab is approved as well [23]. This research addresses the scepticism of physicians and patients and provides a basis for argumentation and reducing the fear of litigation.

#### Adequacy of indicators

When the WSV was introduced, preferential prescribing of VKA was recommended, with DOACs encouraged only for selected indications. Later, a second and lower-ranking recommended drug target within prescribing DOACs (recommended drugs: apixaban, edoxaban) was introduced [6]. However, even after this update, participants still missed their drug targets regarding DOACs. This case demonstrated the dilemma of surveillance systems such as the WSV: when including new drugs, they can be ‘too quick’ by permitting drugs with limited evidence base and high cost or ‘too slow’ by preventing effective and safe new drugs from reaching patients.

Various drugs are available for the therapy of MS. In particular, therapy with glatiramer acetate and interferon beta 1b has long been seen as first-line therapy, and therapy started with them [24, 25]. Accordingly, these substances were also found as recommended drugs within the framework of the WSV, while the monoclonal antibodies alemtuzumab and natalizumab were not [9]. Younger patients with a high number of relapses benefit from early use of a high escalation level [25, 26]. For these cases, monoclonal antibodies, such as natalizumab or alemtuzumab, are recommended by the German guidelines for MS [27]. Physicians who sub-specialise in MS treat many young patients with multiple relapses and/or many patients with advanced MS and, therefore, prescribe a high proportion of drugs not recommended.

#### Lack of knowledge regarding WSV

The impression arose while analysing the interviews that some physicians have been reluctant to familiarise themselves with the regulations of the WSV, prescribing targets, feedback procedures, differences between the previous and current surveillance procedures, etc. This contrasts with the information provided by the KVB in written form, web presentations, and even individual consultations.

#### Faulty beliefs

Despite different postulated mechanisms, bisphosphonates and denosumab show comparable effects on fracture risk [28]. In addition, the fracture risk-reducing effect of bisphosphonates does not seem to correlate with an increase in bone density [29]. Guidelines, therefore, restrict BMD measurements to a few exceptions [30]. Still, participants felt a need to monitor their patients’ BMD, thus producing ‘treatment failures’ [30].

#### Ease and safety of application

Infliximab-biosimilars are recommended by the WSV. However, they are administered intravenously over two hours, and patients have to stay in practice for one to two hours for observation regarding allergic reactions [31–34]. After three infusions, the infusion duration could be shortened to one hour [31–33]. The corresponding adalimumab (Humira®) can be self-administered at home [35]. After approval of a subcutaneous form in 2019 for rheumatic arthritis, since 2020, intravenous infliximab can be switched to subcutaneous infliximab in IBD as well [34]. However, the possibility of switching came to light after conducting our interviews.

Even if most adverse drug reactions occur with low frequency, some physicians experience patients with adverse drug reactions more frequently than others due to small sample fluctuations [36, 37]. This personal experience based on very small samples may lead to distorted beliefs and prescribing, making outlier status more likely. It also shows that drug prescribing is often based on personal experience, which, for many, carries more weight than research evidence.

Gastrointestinal side effects with bisphosphonates should not motivate a switch to denosumab. Often, they result from incorrect intake [38] and can be reduced through better patient education [39]. The parenteral application appears more convenient to some patients. One study with a cross-over design showed compliance to be lower with oral bisphosphonate once a week than with parenteral administration of denosumab every six months [40, 41]. In our view, this underlines the importance of effective patient education.

#### Coping strategies

Referring patients to other physicians was a common practice under previous surveillance procedures based on prescribing costs. Primary care physicians, for example, could ease their prescribing budget by referring patients to specialists with more generous budgets. With the WSV, however, based on preferred drugs and generics, the absolute costs of a prescription are no longer relevant. Some referrals thus seem to arise from not having understood the requirements of the WSV [5, 9, 10]. Under the WSV, only referrals for prescriptions not appearing in the list of preferred drugs have a certain rationale for an individual prescriber, who may thus avoid missing their targets. Referrals exclusively motivated for economic reasons increase the complexity of chronic care, are time-consuming and often costly for the patient, and, because referrals are often accompanied by too little information, they increase the risk of medication errors.

#### Medical information management

The conservativism inherent in the WSV seems to clash with the pharmaceutical industry’s efforts to promote novel treatments, which are usually not available as generic formulations. However, since quotas are considerably below 100%, innovative drugs that do not fulfill the requirements of the WSV can be prescribed.

As the example of denosumab shows, expensive medicines are not automatically better than cheaper ones (see above). Exposure to the advertising of novel drugs by the industry may lead to increased drug costs [42, 43]. A systematic review describes that physicians with low exposure to pharmaceutical promotion show an increased prescribing of generic drugs [43]. Furthermore, this review suggests that primary care physicians with more exposure to pharmaceutical promotion show less adherence to guidelines and prescribe a wider range of drugs [43]. If pharmaceutical representatives are used as a source of information and represent one of the main sources of information, this influences beliefs and the physician’s prescribing behaviour. This will also be the case for pharma-sponsored journals and conferences.

### Surveillance policies

This study examined the prescription of drugs within the framework of the WSV. The physicians are not always sufficiently informed about the requirements, even if they received in-person support from the Bavarian KV.

For prescribing drugs, physicians have to consider not only the indication, comorbidities, and simultaneous treatments but also requests formulated by their patients, expectations by their profession, current guidelines, and the results of clinical trials. All measures addressing the economy of drug prescription thus increase the cognitive and, potentially, the financial burden for prescribing physicians. Since all drugs with a market authorisation in Germany are covered by the SHI, additional measures to contain costs are needed, such as the WSV and comparable surveillance policies. In essence, this leads to bedside rationing [44–46]. A restriction on the range of drugs covered by the SHI has been attempted in the past (‘Positivliste’) but was stopped by the federal government, not least because of pressure from the pharmaceutical industry [47–50]. The only regulation concerning the healthcare system is prices for novel drugs. Only if the joint committee decides that a new drug has benefits compared to established treatments can manufacturers obtain a higher price, which must be negotiated with SHI.

### Implication for the future

The physicians’ evaluation of their practice seems to differ from the feedback provided by the WSV. In this case of cognitive dissonance, it is important to understand and respect physicians’ attitudes and challenges and find solutions for the problems and dilemmas addressed by our interview study.

To understand and support physicians, especially outliers, and still achieve the objectives of the surveillance policies, we have the following suggestions:

1. surveillance policies should be designed and function in a way to avoid unnecessary complexities;
2. information about surveillance and feedback procedures must be available in an easily accessible form;
3. efforts must be made to understand prescribing behaviour, barriers against rational prescribing, and compliance with regulations, such as the WSV, to help develop specific interventions targeted at the mechanisms identified in our study;
4. strengthen drug information and prescribing independent of commercial interests;
5. a system-wide decision regarding the covering of drugs by SHI would be a relief for physicians and reduce the burden of bedside rationing. This, however, will place the onus on central institutions, such as the joint committee and/or the federal government, to introduce appropriate legislation;
6. conflicts of intersectoral (hospital and ambulatory sector) can only be avoided by establishing surveillance procedures for the hospital sector in parallel to ambulatory care; moreover, surveillance should differentiate between first prescribers of problematic drugs and practices issuing repeat prescriptions; and
7. to support physicians, additional information campaigns aimed at the general public and patients should be developed.

There is not enough space to discuss surveillance procedures, indicators (targets), cut-offs etc. Therefore, see our analyses regarding analysing prescribing data [11].

### Strengths and limitations

Participating physicians might predominantly be those physicians, who are interested in the research topic. Although an independent academic institution was asked to conduct the study, potential participants may have feared being unduly charged with inappropriate prescribing behaviour, which may have prevented some from participating. Despite the low number of attendees, this study is relevant to understanding why physicians struggle with their prescribing behaviour and their drug targets. Based on these insights, surveillance policies can be improved, which will result in better prescribing.

To avoid bias and conflict of interest, this study has been conducted by independent, university-based researchers. Nevertheless, preconceptions by members of the research team may have influenced the conduct of interviews and analysis of the text material.

This study only interviewed physicians who had missed their overall targets. For this reason, findings regarding their beliefs and behaviours cannot be generalised to the majority who have met their targets. Moreover, not all specialisation groups were represented in our sample. Still, we think that the beliefs and expectations (mechanisms) identified apply to other clinical areas, sectors and specialisation groups. The dynamic nature of drug targets, surveillance procedures and the drug market present a challenge to research of this kind. This is not necessarily an argument against our results or conclusions but underlines the need to repeat surveys of this kind at regular intervals.

## Conclusion

Only few physicians miss their drug targets and become outliers. We demonstrate mechanisms and challenges in prescribing behaviour (e.g., competing demands). Discussion with study participants also demonstrates the ambiguity of innovation: innovators can be physicians looking for the best available treatments for their patient, but there are also those, who uncritically follow the latest fashion, being victim to pharma marketing. While there are some criticisms of the WSV, there are other mechanisms, which are related to physicians’ behaviours and values (e.g., medical information management). Surveys as reported by us should regularly be conducted to explore reasons for problematic behaviours and develop interventions to improve prescribing.

## Supplementary Information

Supplementary Material 1.

## Data Availability

No datasets were generated or analysed during the current study.
