# Supplementary material for: Surveillance of drug prescribing: why outliers miss their targets – a qualitative study
Source: BMC Health Serv Res. 2025 Jan 3;25:17. doi: 10.1186/s12913-024-12189-0 (PMC11697825; doi:10.1186/s12913-024-12189-0)
Supplement: Supplementary file 1 — Supplementary Material 1. [file 12913_2024_12189_MOESM1_ESM.docx]

**S1: Interview guide**

The interview guide is only intended to provide orientation and preparation. The exact formulations etc. result from the course of the interview and are to be regarded here as examples. Approximately 2 case studies are prepared for each interview and constructed depending on the area of specialisation group.

**Introduction:**

Firstly, we would like to thank you once again for agreeing to take part in our study. Before we start, we would like to introduce ourselves and explain the interview process. We are from the University of Marburg and the University of Erlangen (departments of general and family medicine). As you have already read in the letter, we are interested in the prescribing behaviour of physicians.

We are particularly interested in your personal experiences, opinions, and assessments on various topics. To simplify matters, we have also prepared small case studies. Our project focusses on drug therapy and the prescribing behaviour of physicians. The main aim here is to give a voice to physicians who have not always achieved the targets set by the association of statutory health insurance (SHI)-accredited physicians (KV) in the Bavarian Drug Agreement (WSV). We are interested in the background, challenges, and your approach to drug prescribing.

As you have already read, the interview will last a maximum of 2 hours and will be digitally recorded. We then transcribe the interview and anonymise all data on people, location, or other sensitive information. The information is treated confidentially and ultimately only published as a summarised, analysed data set. Strict care is therefore taken to ensure that no conclusions can be drawn about you personally. Once the study has been completed, the audio files will be deleted.

If there are no questions from you in advance, we can start the interview; at the end of the interview, we have planned further time for questions and suggestions from you.

1. **General information on physician and practical experience**

You have already filled out a short questionnaire (sex, age, specialization group, working experience), but we would like to get to know you and your working environment a little better.

Let us describe your everyday practice life:

- Thinking about your work as (specialisation group), what is the best aspect of your job? What aspects are less enjoyable?
- When you think about your daily work, how can we imagine a typical patient of yours? Can you describe this typical patient to us in a few words?

1. **Role as a physician**

Self-image as a physician:

- We are interested in what you think makes a good physician. So, if you meet young physicians, what would you tell them if they asked what qualities they might need?

🡪 Therefore, we have brought cards with us on which characteristics or behaviours are written. You can look at these at your leisure (pause) - now please take out three cards that you think are important and tell us why.

(Acts economically; Responsible; Strong moral character; Tolerance of uncertainty in decision making; Shared decision making; Works on the current state of science; Leadership skills; Cooperation with colleagues; Critically scrutinising regulations (e.g., health insurance companies); Independence from industrial interests)

🡪 Which terms have you chosen and why?

🡪 Is there any important characteristic that you are missing here?

1. **General prescribing behaviour**

Now it's about therapy options and drug prescriptions.

Example: When a patient comes to your practice, you make a diagnosis, and the patient needs drug-therapy:

- What criteria are important to you when choosing between different drug therapies?

🡪 For example: Mechanism of action; Effect; Recommendation of guidelines; Manufacturer; Prices; Personal experience; Pharmaceutical representatives; Further training; Congresses; Patient wishes; Former financial sections; Concomitant drugs; Comorbidity; Studies

New drugs are regularly authorised on the market, and you must decide whether you want to give them to your patients or not.

- How do you go about this?
- How do you go about selecting the active ingredient or the specific manufacturer?
  - Do you predominantly prescribe active ingredients or concrete drugs with the name of the manufacturer?
  - What impact on your choice has the price of a drug? - Why?
- What comes to mind when you hear the expression ‘the physicians' therapeutic sovereignty’?
  - How do you implement this in everyday life?
- What reasons lead you to refer patients to another specialist?
  - When do you refer for the drug prescription?
  - What kind of prescriptions could be a reason to refer to another specialist?

1. **patient-related prescribing behaviour**

We will now focus a little more on specific patients who come to your practice.

🡪 presenting a case study

We are interested in your approach to this patient. It is not a question of right or wrong, but rather your personal considerations in an area in which there are many different opinions.

- Which drug would you use/prescribe in this case?

We have prepared cards with a selection of active ingredients. Please put them in the order of your preference and explain your decision to us.

- Some drugs are expensive and there is no additional benefit (decision by the federal joint committee (G-BA)). In which case would you use it anyway?

Example: A patient with long-term therapy was in about 6 weeks ago and received a prescription of 100 tablets (dosage: 1 tablet/day). Now he asks for a new prescription.

- How do you handle follow-up prescriptions in your practice?
  - Who prepares the prescription for follow-up prescriptions? (medical assistants/nurses, physician himself?)
  - Do you check whether all tablets may already be used up?
- How do you deal with explicit patient requests for certain drugs or manufactures?
  - How often occur patient requests in practice?
  - What kind of requests are these, for example?
  - How are they dealt with? (example?)

1. **General situation**

Now we would like to know more about your current situation in general.

- How satisfied are you currently with your prescription situation?
  - How do you rate the satisfaction of your colleagues/other specialists with their prescription situation?
- Which prescriptions or regulations have led to difficulties for you so far?
- What would ease prescribing in your opinion?

1. **Bavarian Drug Agreement (WSV)**

In the next section, we would like to focus on the WSV. The WSV probably means a lot more to you than it does to us non-medical professionals. It has set drug targets to be achieved in a wide variety of indication areas. For example, the aim is to prescribe a defined proportion of recommended drugs or generics.

- What experiences have you had with the Bavarian Drug Agreement?
  - Has your prescribing behaviour changed since the beginning of the WSV?
  - Advantages/disadvantages compared to previous surveillance procedures?

Presentation of the paragraphs §12 (1) SGB V and §106 (1) SGB V (Social Security Code):

- What could the development of these paragraphs look like in five to ten years' time?
- What is your general attitude towards the organisations that represent you and other physicians?
- What do you expect from the organisations that represent you and other pyhsicians?

1. **Staying Up-to-Date**

This topic now deals with how you obtain information.

- What sources of information do you use to stay regularly ‘up to date’ on the supply of drugs in your daily practice?
  - Explain ‘regularly’: daily/weekly/monthly
- If you have specific questions about a drug, how do you proceed?
  - Concrete: efficacy, safety, cost-effectiveness - how do you inform yourself?
- What role does it play for you to receive information on drugs from pharmaceutical representatives?

1. **Finalisation**

The last section provides space for other aspects that are still important to you.
As our main aim is to give a voice to physicians who have not always achieved the targets set by the KV, one question is particularly important to us:

- How are you personally doing with it?
- Motivation to work?
- Finally, which aspects would you like to address?
- What was your motivation for participating in our study?
- Perhaps a somewhat provocative question at the end: in your opinion, are the terms ‘cost-effectiveness’ and ‘quality’ compatible in the field of drug therapy?

**Conclusion: Thank you for your participation**

Further questions if possible/necessary:

- How can we imagine discussions with pharmaceutical representatives?
  - Are studies on products presented?
  - Are advantages/disadvantages compared to other products presented?
  - Which drug groups do you focus on the most?
  - What determines whether a pharmaceutical representative can convince you to try the drug?
  - What do you take away from such discussions?
  - Do you gain anything from the discussions?
  - What do such discussions do to you?
- To what extent have you experienced adherence problems with patients regarding the prescription of drugs?
  - What do patients say about the prescriptions? Are there many objections regarding the choice?
  - Do patients refuse to take the drugs if you do not prescribe what they want?
  - Are drugs accidentally mixed up, which can lead to incidents or even emergencies?
